# Volume and antimicrobial activity of secretions of the uropygial gland are correlated with malaria infection in house sparrows

**DOI:** 10.1186/s13071-016-1512-7

**Published:** 2016-04-25

**Authors:** Sergio Magallanes, Anders Pape Møller, Luz García-Longoria, Florentino de Lope, Alfonso Marzal

**Affiliations:** Departamento de Anatomía Biología Celular y Zoología, Universidad de Extremadura, Avda. de Elvas s/n, E-06006 Badajoz, Spain; Laboratoire d’Ecologie, Systématique et Evolution, CNRS UMR 8079, Université Paris-Sud, Bâtiment 362, F-91405 Orsay Cedex, France

**Keywords:** Antimicrobial activity, Flow cytometry, Haemosporidian parasites, *Passer domesticus*, Preen gland

## Abstract

**Background:**

Animals have developed a wide range of defensive mechanisms against parasites to reduce the likelihood of infection and its negative fitness costs. The uropygial gland is an exocrine gland that produces antimicrobial and antifungal secretions with properties used as a defensive barrier on skin and plumage. This secretion has been proposed to affect the interaction between avian hosts and their ectoparasites. Because uropygial secretions may constitute a defense mechanism against ectoparasites, this may result in a reduction in prevalence of blood parasites that are transmitted by ectoparasitic vectors. Furthermore, other studies pointed out that vectors could be attracted by uropygial secretions and hence increase the probability of becoming infected. Here we explored the relationship between uropygial gland size, antimicrobial activity of uropygial secretions and malaria infection in house sparrows *Passer domesticus*.

**Methods:**

A nested-PCR was used to identify blood parasites infection. Flow cytometry detecting absolute cell counting assessed antimicrobial activity of the uropygial gland secretion

**Results:**

Uninfected house sparrows had larger uropygial glands and higher antimicrobial activity in uropygial secretions than infected individuals. We found a positive association between uropygial gland size and scaled body mass index, but only in uninfected sparrows. Female house sparrows had larger uropygial glands and higher antimicrobial activity of gland secretions than males.

**Conclusion:**

These findings suggest that uropygial gland secretions may play an important role as a defensive mechanism against malaria infection.

## Background

Parasites are ubiquitous and the most abundant organisms on Earth [1, 2]. They cause harmful effects on their hosts and negatively influence different host fitness components, such as growth [3], survival [4], fecundity [5] and reproductive output [6, 7]. Because of these effects exerted by parasites, animal hosts have developed a wide range of defensive mechanisms in order to reduce the likelihood of infection and/or its negative effects [8, 9]. These mechanisms include natural resistance to infection such as physical barriers to invading pathogens or high-density lipoproteins in human serum destroying trypanosomes [8, 10], nonspecific immune responses such as generation of oxidative products by the phagocytes to destroy microbes [11], specific immune responses such as the production of a variety of antibodies that bind to specific pathogens [12], or behaviours aiming to control exposure to parasites [13, 14].

Avian malaria and related haemosporidian parasites are among the most pathogenic species of poultry and wild birds [15]. These widespread organisms cause detrimental effects on life history of their avian hosts by reducing survival [16, 17], reproductive success [7, 18, 19] and body condition [20, 21]. They show a complex life-cycle [15], in which the presence of a vector (biting midges, black flies, louse flies and a large number of mosquito species) is needed for transmission of the disease [15, 22].

The uropygial gland (also called preen gland) is an exocrine gland of birds secreting waxes with antimicrobial and antifungal properties that is smeared on the plumage during preening and hence acting as defensive barrier of skin and plumage [23–27]. This secretion has been proposed to play an important role in host-parasite relationships because it may affect the interaction between birds and their vectors [28, 29]. However, the possible role of the uropygial gland in protection against haemosporidian parasites still remains unclear.

Uropygial secretions may prevent birds from acquiring blood parasite infection. Mammalophilic and ornithophilic mosquitoes and other ectoparasites act as disease vectors that rely on different cues to locate their potential blood hosts. Host finding by vectors is largely driven by visual stimuli [30], exuded heat resulting from metabolic activity of hosts [31] and odorant and volatile organic chemicals produced by skin and plumage bacteria that are emitted by individual hosts [32–34]. Therefore, the antimicrobial activity of the uropygial secretion from birds can decrease feather and skin microbiota and hence reduce the emission of chemical cues used by haemosporidian vectors. This should minimize the likelihood of being infected with these blood parasites. In addition, compounds isolated from secretions of the uropygial gland have an insecticidal effect [35] and can act as ectoparasite repellents [36]. Therefore, these secretions may also decrease the probabilities of haemosporidian infection.

Alternatively, antiparasite defences may negatively affect hosts when such defences become attractants for parasites or vectors. Following this idea, several studies have indicated that uropygial secretions may be used by haemosporidian vectors to locate potential hosts and infect them. For example, Lowther & Wood reported that some species of black flies were strongly attracted to uropygial gland extracts from common loon (*Gavia immer*) [37]. Similarly, Fallis & Smith pinpointed the uropygial gland as one of the main sources of black fly attraction to common loons [28]. Moreover, Bennett et al. successfully used ether extract of the uropygial gland of the common loon to attract simulids [38]. More recently, Russell & Hunter showed that traps baited with uropygial secretion from American crows *Corvus brachyrhynchos* captured more *Culex* mosquitoes, one of the main avian malaria vectors [15], than unbaited blank control traps [29].

Here we investigate these two hypotheses on the role of uropygial gland secretions on haemosporidian infections in the house sparrow *Passer domesticus*, one of the most ubiquitous hosts for avian malaria [39]. First, we analyse the relationship between haemosporidian infection and the size of the uropygial gland, a reliable measure of the volume of produced secretion [40]. If the properties of uropygial secretions can minimize the attraction of vectors to birds, then we expect a lower prevalence of haemosporidian infection in individual birds with larger uropygial glands. In contrast, if haemosporidian vectors are attracted to the uropygial secretion, then we should expect that sparrows with larger uropygial glands were more prone to infection with haemosporidians than birds with smaller glands. Secondly, we explore the relationship between antimicrobial capacity of secretions from the preen gland and blood parasite infection. If haemosporidian infection can be mediated by odour stimuli produced by skin and plumage bacteria attracting insect vectors, we should expect that sparrows with higher antibacterial activity of their gland secretions would have a lower probability of being infected with these blood parasites.

## Methods

### Study sites and sample collection

The study was carried out in a rural (38°39'N, 7°13'W) and an urban population (38°53'N, 7°00'W) near Badajoz, southwest Spain between November - December 2014. We captured 222 adult house sparrows (55 sparrows in one urban site, 167 sparrows in one rural site) in five days during the same week with mist-nets and recorded their body mass with a digital balance to the nearest 0.1 g. We measured tarsus length with a digital calliper to the nearest 0.01 mm. We used body mass and tarsus length to calculate scaled body mass index [41], which is a reliable estimate of animal physical condition [42]. Each individual was individually identified with a numbered metal ring and sexed according to Svensson et al. [43]. One microcapillary of blood (70 μl) was obtained from the brachial vein of each individual and stored in 500 μl of SET buffer (0.15 M NaCl, 0.05 Tris, 0.001 M EDTA, pH 8.0) until DNA extraction.

### Molecular detection of blood parasite infections

Haemosporidian parasites (*Plasmodium* spp. and *Haemoproteus* spp.) were detected from blood samples using molecular methods [44, 45]. DNA from the avian blood samples was extracted in the laboratory using a standard chloroform/isoamylalcohol method [46]. Diluted genomic DNA (25 ng/μl) was used as a template in a polymerase chain reaction (PCR) assay for detection of the parasites using nested-PCR protocols described by Waldenström et al. [45]. The amplification was evaluated by running 2.5 ml of the final PCR on a 2 % agarose gel. All PCR experiments contained one positive control and one negative control for every eight samples. In the very few cases of negative controls showing signs of amplification (never more than faint bands in agarose gels), the whole PCR-batch was run again to make sure that all positives were true.

### Volume of the uropygial gland secretion

We recorded length, height and width of the uropygial gland with a digital calliper with a precision of 0.01 mm. Uropygial gland volume was estimated as the product of length, height and width [47], which is positively related to the volume of uropygial gland secretions [26, 40, 48]. Because the uropygial gland is a soft tissue [26, 48], we measured the three dimensions of uropygial gland three times to calculate repeatability [26, 49, 50].

We also extracted all the secretion available in the uropygial papilla immediately after capture of 44 individuals from the same location, following the extraction protocol described by Martín-Vivaldi et al. [48]. Briefly, we first washed the uropygial gland and surrounding skin with a cotton swab soaked in ethanol to reduce the risk of contamination of the secretion. After evaporation of the alcohol, the papilla was softly pressed with a finger to expel the secretion and transfer it into a micro-capillary tube until the papilla was empty. Immediately after extraction we estimated the volume of the secretion in the filled capillary tube with a digital calliper with an accuracy of 0.01 mm. The extracted secretion was transferred to a sterile Eppendorf vial and kept at about 4 °C in a portable icebox, and stored in the laboratory at -20 °C during the next 4 h until analyses of anti-microbial activity.

### Bacterial growth and antimicrobial activity of uropygial gland secretions

The pellet of *Staphylococcus epidermidis* (ATCC ® CRM – 12228™) was re-suspended in 6 ml of Luria - Bertani (LB) media to an OD 0.4–0.6 and incubated at 37 °C with shaking for 24 h. The bacterial suspension was then centrifuged in a Microfuge (Beckman Coulter) for 6 min at 2000 *g*. After discarding the supernatant, the bacterial pellet was re-suspended carefully in 20 ml LB solution. A total of 200 μl per well of bacterial suspension were then dispensed in a 96-well plate. Uropygial secretions were diluted 1:1 in dimethylsulfoxide (DMSO); 1 μl of the uropygial secretion diluted with DMSO was added to the bacteria culture in each well. Four wells on a 96-well plate with bacteria solution were not added with uropygial secretions but with 1 μl of DMSO, as they were used as controls of bacterial growth. After culture incubation for bacterial growth at 37 °C for 24 h, the 96-well plate was covered and centrifuged in a plate centrifuge (Selecta, Spain) for 5 min at 2000 *g* and the pellet were re-suspended in 200 μl of PBS at a final concentration of 0.6 μg/ml. Samples were then incubated at 37 °C for 30 min in the dark with shaking. Flow cytometry detecting absolute cell counting assessed antimicrobial activity of the uropygial gland against *S. epidermidis* secretion. This technique is a rapid, accurate and highly reproducible methodology used in clinical microbiology to monitor antimicrobial activity [51]. A total of 50 μl of the cell suspension from each well was acquired using a MACSQuant® X (Miltenyi Biotec) flow cytometer that allows absolute cell counting. Antimicrobial activity was evaluated by comparison of cell counting (bacterial growth) in wells with presence or absence (controls) of uropygial secretion.

### Statistical procedures

Repeatability of uropygial gland measurements was calculated following the approximate Gaussian LMM using REML estimation (R_M(REML)_) described by Nakagawa & Schielzeth [52]. We performed Shapiro-Wilk test for normality of distribution of data and used general linear models (GLM) to investigate the relationship between sex, locality (i.e. environmental variation), scaled body mass index, infection status (uninfected or infected) and the two-way interactions between sex and scaled body mass index, between infection status and scaled body mass index, and between infection and locality, on the uropygial gland volume. We used Pearson correlation test to determine the strength of association between uropygial gland volume and scaled body mass index regarding to sex and infection. We also used a GLM to evaluate the correlation between sex, uropygial gland volume, scaled body mass index and infection status (uninfected or infected) on the antimicrobial activity of the uropygial secretion. All analyses were performed using R version 3.2.2 [53] and JMP [54].

### Ethics statement

Methods were evaluated and approved by Institutional Commission of Bioethics of University of Extremadura (CBUE 49/2011). All the experiments comply with the current laws of Spain, where the experiments were performed.

## Results

We analysed 222 blood samples from house sparrows in search of blood parasites. A total of 74 % (165 individuals) were uninfected and 26 % (57) were infected with blood parasites (13.5 % of sparrows infected in the rural location, 49.1 % of sparrows infected in the urban location).

The uropygial gland volume and the antimicrobial activity of the uropygial secretion showed a normal distribution (Shapiro-Wilk normality test; Uropygial gland volume: *N* = 222, *W* = 0.923, *P* > 0.001; antimicrobial activity: *N* = 44, *W* = 0.943, *P* = 0.030). We found a high repeatability between measurements of length, width and height of uropygial gland (all *R* > 0.81 and *P* < 0.05).

Prevalence of haemosporidian parasites, sex and locality explained significant variation in size of the uropygial gland. In contrast, scaled body mass index was not significantly correlated with size of the uropygial gland (Table 1). Specifically, the volume of the uropygial gland was larger in uninfected than in infected house sparrows [mean uropygial gland volume (standard deviation, SD): uninfected = 171.84 mm^3^ (43.22); infected = 161.03 mm^3^ (34.33)]. Furthermore, the volume of the uropygial gland differed between the sexes, with females having larger glands than males [mean uropygial gland volume (SD): females = 171.57 mm^3^ (40.01); males = 167.36 mm^3^ (42.26)]. Moreover, the volume of the uropygial gland differed significantly among localities, with house sparrows living in an urban site having larger uropygial glands than sparrows in a rural site [(mean uropygial gland volume (SD): urban sparrows = 184.75 mm^3^ (45.28); rural sparrows = 163.90 mm^3^ (38.70)].

**Table 1:** Factors explaining the variation in volume of the uropygial gland in house sparrows. Scaled body mass index, haemosporidian infection, sex, locality and the two-way interactions between sex and scaled body mass index, between infection status and scaled body mass index and between infection and locality were included in the analysis as predictor variables. Sample size was 222 individuals

Independent variable	Square-sum III	*DF*	*F*	*P*
Scaled body mass index	4695.89	1	0.190	0.7176
Infection	3787.71	1	4.082	0.046
Sex	5574.75	1	4.122	0.036
Locality	14282.25	1	10.464	< 0.001
Sex × Scaled body mass	5061.31	1	4.084	0.045
Infection × Scaled body mass	4695.89	1	4.354	0.027
Infection × Locality	1153.53	1	0.845	0.359

**Fig. 1:**
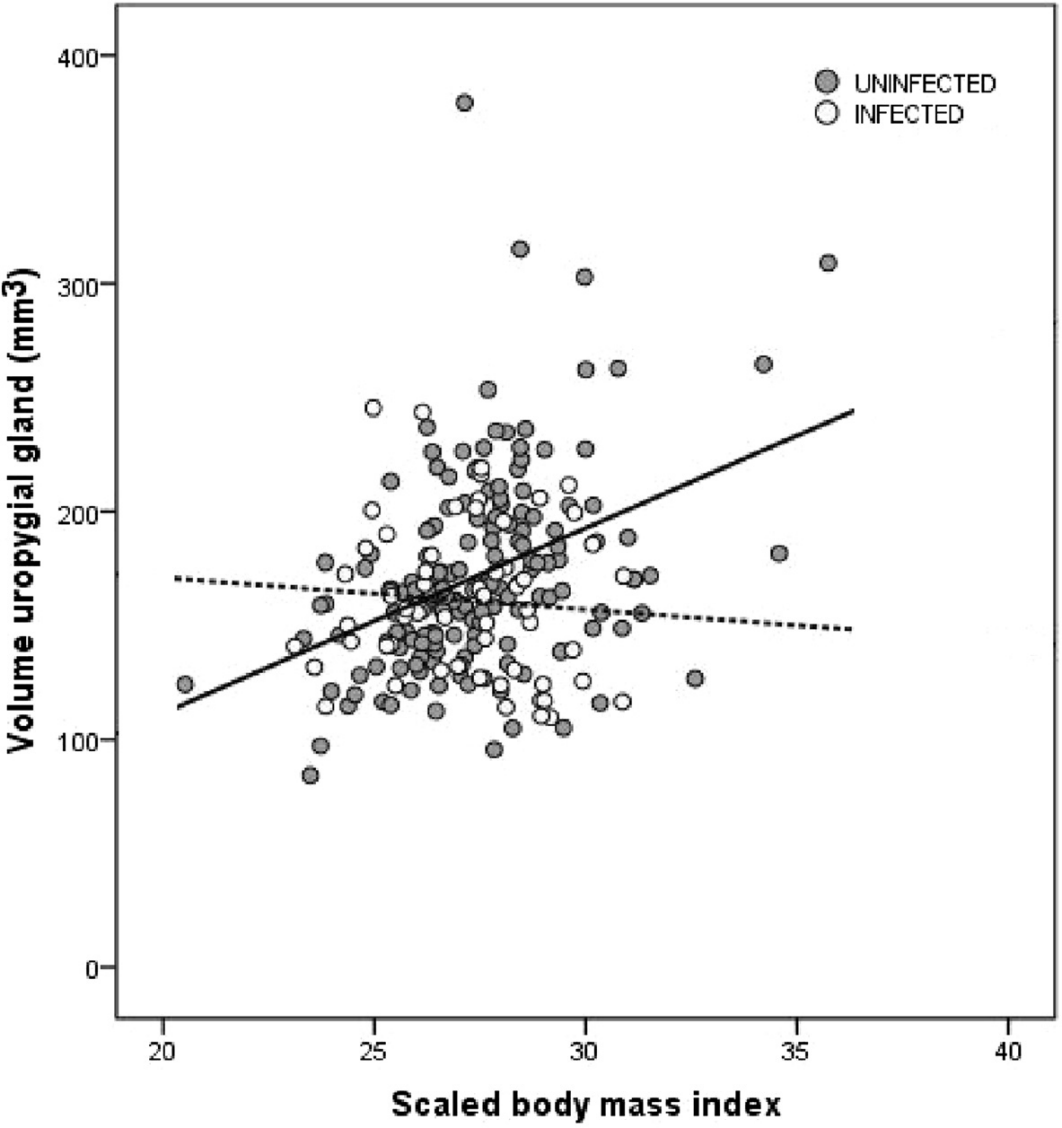
Scatterplot showing the relationship between the volume of the uropygial gland (mm^3^) and scaled body mass index in haemosporidian infected (white circles, dotted line, *N* = 57) and uninfected house sparrows (grey circles, solid line, *N* = 165)

The relationship between the volume of the uropygial gland and scaled body mass index varied with haemosporidian infection (Table 1; Fig. 1). Specifically, there was a positive relationship between gland size and scaled body mass index in uninfected house sparrows (*r* = 0.392; *P* < 0.001), while there was no significant relationship in individuals infected with haemosporidians (*r* = - 0.077; *P* = 0.568). We also found a positive relationship between gland size and scaled body mass index in both males (*r* = 0.271; *P* = 0.002) and females (*r* = 0.349; *P* = 0.001).

In a second GLM we examined if antimicrobial activity of the uropygial secretion varied with sex, haemosporidian infection, scaled body mass index and uropygial gland volume. The estimate of antimicrobial activity of the uropygial gland varied with haemosporidian infection and sex (Table 2). Specifically, antimicrobial activity of uropygial secretion was significantly higher in uninfected than in infected birds [mean antimicrobial activity (SD): infected = 11.08 (9.76); uninfected = 20.39 (15.81)]. Finally, uropygial gland secretions from female sparrows had a slightly higher antimicrobial activity than secretions from male sparrows, although non-significantly so [mean antimicrobial activity (SD): females = 20.79 (16.66); males = 16.18 (13.73)].

**Table 2:** Factors explaining the variation in antimicrobial activity of the uropygial gland in house sparrows. Scaled body mass index, haemosporidian infection, sex and uropygial gland volume were included in the analysis as predictor variables. Sample size was 44 individuals

Independent variable	Square-sum III	*DF*	*F*	*P*
Scaled body mass index	1.38	1	2.011	0.1756
Infection	-2.43	1	5.850	0.0196
Sex	1.75	1	2.947	0.0872
Gland volume	-5.19	1	0.297	0.4968

## Discussion

Uropygial secretions have been hypothesized to play an important role in the interaction between birds and haemosporidian parasites because it can affect the interaction between hosts and their vectors. Although the results from some studies do not support a potential role of avian uropygial gland secretions in attracting haemosporidian vectors [55], other studies have indicated that preen oil secretions may constitute a defence mechanism against ectoparasites and thus avoidance of blood parasite infections [56]. In contrast, other studies suggest that haemosporidian vectors may be attracted by uropygial secretions [28, 29]. Here we explored the relationship between uropygial gland size, antimicrobial activity of uropygial secretions and malaria infection in house sparrows. The main findings of this study were that (i) uninfected house sparrows had larger uropygial glands and higher antimicrobial activity in uropygial secretions than infected house sparrows; and (ii) female house sparrows had larger uropygial glands and higher antimicrobial activity than males. We briefly discuss these results.

More than 200 blood samples from house sparrows were analysed in search of blood parasites. A total of 26 % of house sparrows were infected with haemosporidian parasites. Similar haemosporidian prevalence during winter has been found in previous studies of house sparrows from the same area [57].

Uninfected house sparrows had larger uropygial glands than infected house sparrows, suggesting that the uropygial gland and its secretions may constitute defences against ectoparasites, and thus against haemosporidian infection. Thus, González [58] have recently shown that ectoparasite richness and ectoparasite burden is negatively related to the mass of the uropygial gland in rock ptarmigan (*Lagopus muta*). Moreover, other studies have shown that individuals with larger glands, and, therefore, able to produce more uropygial secretions, have lower prevalence of ectoparasites [48, 59]. We propose different mechanisms that may explain this protection by preen secretions against ectoparasites. First, uropygial secretions may act as a physical barrier against vectors by reducing their mobility on the bird’s plumage or skin [56]. Secondly, uropygial secretions could act as an insecticide and kill ectoparasites by covering the surface of the parasite or blocking their spiracles [35]. Finally, preen secretions may be associated with noxious or repellent odours that possibly affect ectoparasites, as has been shown in some species of birds [60]. We also found a positive and significant relationship between gland size and scaled body mass index in uninfected house sparrows, while the volume of the uropygial gland was not significantly related to scaled body mass index among malaria infected sparrows. These findings suggest that development of the uropygial gland is costly and may impair energetic demands [61, 62]. Thus, only individuals in prime condition (uninfected sparrows) should be able to invest in anti-microbial defence without compromising other fitness-related traits.

The antimicrobial and anti-fungal properties of uropygial secretions may benefit birds by avoiding haemosporidian infection. Consistently, we showed that the antimicrobial activity of the uropygial gland in uninfected house sparrows was higher than that of gland secretions from infected birds. As far as we are aware, this is the first study showing a relationship between antimicrobial activity of uropygial secretions and haemosporidian infection. We hypothesize that this relationship may be mediated by a decrease in olfactory stimuli for insect vectors caused by preen secretions. Accordingly, it has been shown that the odours emanating from gland secretions and the microflora of the skin may play an important role in attraction of malaria vectors in humans [63–65]. Birds harbour a great diversity of microbes on feathers and skin, which may be involved in the production of chemical attractants for haemosporidian vectors like *Culex* spp. and simulids [28, 37, 66]. Hence, the elimination of bacteria and fungi from feathers and skin by uropygial secretions could decrease vector attraction and thus minimize the likelihood of becoming infected with haemosporidians. Following this idea, uropygial secretions may affect different strains of parasitic bacteria and fungi in different ways. For example, it has been shown that volatile compounds (saturated fatty acids, benzaldehyde and phenol, among others) from uropygial gland secretions provide strong antimicrobial action in birds [67]. Similarly, Jacob et al. showed that uropygial secretions from birds of the order Pelecaniformes had an antagonist effect on fungal dermatophytes and an antibacterial effect on Gram-positive bacteria [68]. Moreover, Law-Brown experimentally showed activity of uropygial secretions against 13 pathogenic bacterial strains from the genera *Salmonella*, *Staphylococcus* and *Streptococcus* [69]. Additionally, it has been proposed that pathogenic bacteria and fungi may be controlled through the synthesis of bacteriocins and other antimicrobial actions of certain symbiotic bacteria that live in the preen gland [23–25, 70].

Female house sparrows have larger uropygial glands than male sparrows. In agreement with our results, previous studies of birds have shown that females have larger glands than males [40, 48, 59]. Moreover, the antimicrobial activity of preen secretions from female sparrows was slightly higher than that of males. Bacteria and other pathogens in the nest are known to be one of the main factors affecting egg survival and hatching success [71–73]. Because in many species of birds females spend more time in nests during incubation and nestling periods than males [74, 75], larger uropygial glands and/or higher antibacterial capacities may lead to a fitness advantage in terms of higher hatching success [76]. Alternatively, several studies have shown that females are more infected with haemosporidian parasites than males [77, 78], suggesting a female bias in exposure to the vectors [79]. Because time spent in the nest can increase the risks of becoming infected with haemosporidian parasites [15], larger uropygial glands and higher antimicrobial activity may provide females with a higher protection against haemosporidian vectors.

Birds are expected to modify their investment in defensive traits (uropygial gland) in response to differences in exposure to microorganisms [80]. Animals living in cities are exposed to more pathogenic diseases than their rural counterparts [81, 82]. In agreement with these expectations, we found a significant difference in size of uropygial glands between populations. However, our results rely on only one single comparison, and hence, we need more replicates of uropygial gland sizes from other urban and rural populations of house sparrows in order to draw firm conclusions.

## Conclusion

In conclusion, the size of the uropygial gland and the antimicrobial activity of its secretions varied with haemosporidian infection and sex in house sparrows. These findings suggest that uropygial glands may be involved in defensive mechanisms against malarial infections under natural conditions. Further experimental studies could help improve our understanding of this bird-parasite interaction. They may also help to test if uropygial secretions may have properties that reduce or eliminate the risk of malarial infection.
